# 

**Published:** 1902-01-04

**Authors:** 


### NOTICE TO CORRESPONDENTS.

All MSS., Letters, Books for Review, and other matters intended for the Editor should be addressed THE EDITOR, "THE HOSPITAL," THE HOSPITAL BUILDING, 28 & 29 SOUTHAMPTON STREET, STRAND, W.C.
The Editor cannot undertake to return rejected MSS., even when accompanied by stamped directed envelope.

**Notice to Advertisers and Subscribers.**

All Advertisements, Orders for Copies of THE HOSPITAL, and Business Communications should be addressed to **THE MANAGER (Not to the Editor),** THE HOSPITAL, 28 & 29 Southampton Street, Strand, London, W.C.

The Cost of Subscription, post free, is as follows:—Per week, 2½d.; per quarter, 2s. 8d.; per half-year, 5s. 3d.; per annum, 10s. 6d. Foreign:—Per annum, 15s. 2d., payable, in all cases, in advance.

**NOTICE.—Vol. XXX. of "The Hospital" (April 1901, to September 1901), in handsome cloth binding can now be obtained at the office of this Journal, price, 7s. 6d. Cases for binding the Numbers contained in this Volume 1s. 6d. Index to Vol. XXX., 2½d., post free.**

**The Monthly Part for January is now ready. Price 6d., by post 8d.**

### The Student of Medicine.

### APPOINTMENTS.

**W. A. H. Barrett**, L.R.C.P.Lond., L.R.C.S.Edin., appointed Officer of Health for the Shire of Mansfield, Victoria. **James Boag**, M.B., C.M.Glasg., appointed Certifying Factory Surgeon to the Wishaw District of Lanarkshire. **C. W. Bruce**, M.B.Melb., appointed Government Medical Officer and Vaccinator at Cudelo, New South Wales. **C. M. Coates**, L.R.C.P., L.R.C.S.Edin., L.F.P.S.Glasg., appointed Certifying Factory Surgeon for the Creech St. Michael District of Somerset. **Joseph Cookson**, M.B.Melb., appointed Officer of Health for the Shire of Wimmera, Victoria. **Joseph P. Daly**, L.R.C.S., L.R.C.P.Irel., appointed Certifying Factory Surgeon for the Monasterevan District, County Kildare. **Neville R. Howe**, F.R.C.S.Eng., appointed Visiting Surgeon to the Goal at Orange, New South Wales. **Edward A. Johnson**, M.B., B.S.Adel., appointed Honorary Assistant Physician to the Adelaide Hospital. **George Jubb**, M.D.Glasg., appointed Extra Physician to the Glasgow Central Dispensary. **James F. Lovegrove**, M.R.C.S.Eng., L.S.A., appointed Government Medical Officer and Public Vaccinator at Rylstone, New South Wales. **T. H. Parke**, L.R.C.P.Edin., M.R.C.S.Eng., appointed Certifying Factory Surgeon for the Tideswell District of Derbyshire. **Charles Porter**, M.D., R.U.I., B.Ch., appointed Medical Officer of Health to the Municipality of Johannesburg. **D. R. Price**, M.B., C.M.Edin., appointed Medical Officer for the South District of the Llandilofawr Union. **A. Macgregor Rose**, M.B., Ch.B., D.P.H.Aberd., appointed Acting Government Medical Officer No. 4 District, Antigua, British West Indies. **R. Hamilton Russell**, F.R.C.S.Eng., appointed Surgeon to In-patients at the Alfred Hospital, Melbourne, Victoria. **Wm. Atkin Thompson**, M.R.C.S. (Eng.), L.R.C.P. (Lond.), appointed House Surgeon to the Rochdale Infirmary. **E. Paget Thurston**, M.D.Cam., M.R.C.S.Eng., appointed District Medical Officer at Lawlers, West Australia. **Hunter F. Tod**, M.A., M.B., B.C.Cantab., F.R.C.S.Eng., appointed Assistant Aural Surgeon to the London Hospital. **G. A. Upcott-Gill**, M.R.C.S.Eng., L.R.C.P.Lond., appointed Assistant Medical Officer to the St. Pancras Infirmary. **H. B. Wilson**, M.R.C.S.Eng., appointed Government Medical Officer and Vaccinator at Germanton, New South Wales. **R. G. Worger**, M.R.C.S., L.R.C.P.Lond., appointed Certifying Factory Surgeon for the Radstock District, County Somerset.

